# Tile-Based Two-Dimensional Phase Unwrapping for Digital Holography Using a Modular Framework

**DOI:** 10.1371/journal.pone.0143186

**Published:** 2015-11-24

**Authors:** Georgios C. Antonopoulos, Benjamin Steltner, Alexander Heisterkamp, Tammo Ripken, Heiko Meyer

**Affiliations:** 1 Biomedical Optics Department, Laser Zentrum Hannover e.V., Hanover, Germany; 2 Institute of Quantum Optics, Gottfried Wilhelm Leibniz Universität Hannover, Hanover, Germany; Shenzhen institutes of advanced technology, CHINA

## Abstract

A variety of physical and biomedical imaging techniques, such as digital holography, interferometric synthetic aperture radar (InSAR), or magnetic resonance imaging (MRI) enable measurement of the phase of a physical quantity additionally to its amplitude. However, the phase can commonly only be measured modulo 2*π*, as a so called wrapped phase map. Phase unwrapping is the process of obtaining the underlying physical phase map from the wrapped phase. Tile-based phase unwrapping algorithms operate by first tessellating the phase map, then unwrapping individual tiles, and finally merging them to a continuous phase map. They can be implemented computationally efficiently and are robust to noise. However, they are prone to failure in the presence of phase residues or erroneous unwraps of single tiles. We tried to overcome these shortcomings by creating novel tile unwrapping and merging algorithms as well as creating a framework that allows to combine them in modular fashion. To increase the robustness of the tile unwrapping step, we implemented a model-based algorithm that makes efficient use of linear algebra to unwrap individual tiles. Furthermore, we adapted an established pixel-based unwrapping algorithm to create a quality guided tile merger. These original algorithms as well as previously existing ones were implemented in a modular phase unwrapping C++ framework. By examining different combinations of unwrapping and merging algorithms we compared our method to existing approaches. We could show that the appropriate choice of unwrapping and merging algorithms can significantly improve the unwrapped result in the presence of phase residues and noise. Beyond that, our modular framework allows for efficient design and test of new tile-based phase unwrapping algorithms. The software developed in this study is freely available.

## Introduction

The ability to measure the phase of a complex physical signal is a key advantage of imaging methods such as digital holography [1], interferometric synthetic aperture radar (InSAR) [2], or magnetic resonance imaging [3]. However, due to the 2*π* periodicity of a phase map, the resulting measurement typically yields the true phase modulo 2*π*. Such a phase map is called *wrapped* and will show distinctive jumps (wraps) of magnitude 2*π*. From a mathematical point of view this is a consequence of the inverse tangent operation that is typically involved in retrieving the phase of a complex signal. Phase unwrapping is the process of removing the 2*π* ambiguity from the wrapped phase map. This process is trivial for the one dimensional case, but it becomes extremely challenging in two dimensions, even more so when measurement errors deteriorate the quality of the wrapped phase map [4]. Phase unwrapping has been studied for decades, yet due to a growing number of applications, it remains an active area of research until today [5–9].

Phase unwrapping algorithms can be classified in several ways. We follow Harráez *et al.* by dividing phase unwrapping algorithms into three major categories: Global algorithms, path following algorithms, and region-based algorithms [10]. Global algorithms typically work by minimizing a cost function that takes all pixels of the wrapped phase map into account [11]. Solutions are obtained based on transform methods [12–14], Bayesian estimation [7, 15, 16], or network optimization [4, 17, 18]. Path following algorithms unwrap the phase map by detecting 2*π* phase jumps between neighboring pixels along a path. These algorithms operate by using simple linear paths [6], by using sophisticated branch-cut algorithms [19–22], or by choosing an unwrapping path based on a quality criterion [10, 23]. Region-based algorithms work by dividing the phase map into subregions. Phase unwrapping is first performed on subregions and unwrapped regions are grown or merged gradually [24–26].

Tile-based algorithms are a special case of the region-based algorithms [25, 27–30]. The phase map is tessellated into rectangular subregions called tiles that are processed in two steps: First, tiles are unwrapped individually and second, tiles are merged to a continuous phase map. This approach has some very appealing properties, most notably that the tile unwrapping step can be implemented computationally efficiently by parallelization. However tile-based algorithms are prone to error propagation in case of failed unwraps of single tiles as well as phase residues. In this paper we developed a modular C++11 framework for tile-based phase unwrapping. By casting the tile-based approach into a modular framework we were able to implement different tile unwrapping and merging algorithms. Next to established algorithms we also implemented original versions of tile unwrapping and merging strategies. We then compared various combinations of unwrapping and merging algorithms to existing approaches, both tile-based and pixel-based. We could show that our algorithms can improve the result of phase unwrapping in the presence of noise and residues. Our modular software framework enabled us to easily combine different tile unwrapping and merging algorithms for the quantification of the resulting phase unwraps. It was designed as a tool for developing and testing tile-based phase unwrapping algorithms, thus ease of use and readability are emphasized over execution speed. While algorithms were implemented efficiently, the modularity of the framework does introduce an overhead that results in a speed penalty. The software developed for this study is fully documented and freely available as open source software. The source code, a precompiled binary and an ImageJ Plugin are accessible using the GitHub repository https://github.com/gc-ant/digiholo2D.

### Mathematical formalism

Here we will give a brief introduction to the notation and the mathematical formalism used throughout this paper, for detailed overviews see [11, 31]. The two-dimensional unwrapped phase distribution, which is a priori unknown, will be designated with *ϕ*_*u*_(*x*, *y*). A wrap operator W is introduced to obtain the corresponding wrapped phase map *ϕ*_*w*_(*x*, *y*):

$$\begin{array}{c} \phi_{w}\left(x,y\right):=W\phi_{u}\left(x,y\right):=\phi_{u}\left(x,y\right)-2\pi n\left(x,y\right)\text{,}\;\text{s.t.}\;\phi_{w}\left(x,y\right)\in \left[-\pi ,\pi \right),n\left(x,y\right)\in Z. \end{array}$$ (1)

The wrap operator adds a signed integer multiples of 2*π* to the pixels of the unwrapped phase map so that each pixel gets shifted to the interval [−*π*, *π*). Phase unwrapping is the problem of obtaining this integer jump map from the wrapped phase with the aim of obtaining the unwrapped (physical) phase map. The true physical phase map need not be a continuous function but it can be piecewise continuous. In this paper we assume that the phase jumps at locations of discontinuities are of magnitude less than *π*. This assumption holds for smoothly varying objects such as cells or appropriate technical surfaces when imaged using digital holography [32–37]. Furthermore we require that discrete sampling to a grid (*x*_*i*_, *y*_*j*_) is performed such that the *Itoh condition* is satisfied [6]:

$$\begin{array}{c} -\pi <\phi_{u}\left(x_{i},y_{j}\right)-\phi_{u}\left(x_{m},y_{n}\right)<\pi \;\forall \;\text{next}\;\text{neighbors}\;\left(x_{i},y_{j}\right),\left(x_{m},y_{n}\right). \end{array}$$ (2)

Under the assumptions above, the solution to the phase unwrapping problem is unique [31].

An important artifact of quantitative phase imaging are so called phase residues, which can be understood as follows: A true phase map *ϕ*_*u*_ of a complex physical quantity is a scalar function. Therefore the integral of the gradient $\vec{\nabla }\phi_{u}$ along any closed loop must evaluate to zero and the integration is path independent. This implies that for any closed loop along a corresponding wrapped phase map $\phi_{w}=W\phi_{u}$ the number of positive 2*π* jumps between adjacent pixels will equal the number of negative jumps. This is true, if the non-wrapped phase map is either continuous or its discontinuities are of magnitude less than 2*π* and it is sampled according to the Itoh condition [38]. Real wrapped phase maps, however, will often violate this condition at sites termed *residues*[19]. For further illustration of residues refer to S2 Fig. The problem of unwrapping a wrapped phase distribution with residues does not have a unique solution.

### Tile-based phase unwrapping

Let the two-dimensional wrapped phase map *ϕ*_*w*_(*x*, *y*) be given on a discrete rectangular pixel raster of width *N*_*X*_ and height *N*_*Y*_, so that *x*_*i*_, *i* = 1, …, *N*_*X*_ and *y*_*j*_, *j* = 1, …, *N*_*Y*_. The basic idea of tile-based phase unwrapping is to tessellate the wrapped phase map into *N*_*τW*_ × *N*_*τH*_ rectangular tiles *τ*_*w*, *h*_ with indices *w* = 1, …, *N*_*τW*_ and *h* = 1, …, *N*_*τH*_ [25, 27–30]. After that, phase unwrapping is performed in two consecutive steps:

1. Tile unwrapping: Each tile *τ*_*w*, *h*_ is unwrapped individually. This step can, in principle, be performed for all tiles simultaneously.
2. Tile merging: In this step, tiles are merged to a continuous surface using a *merging algorithm* by adding integer multiples of 2*π* to entire tiles.

**Fig 1:**
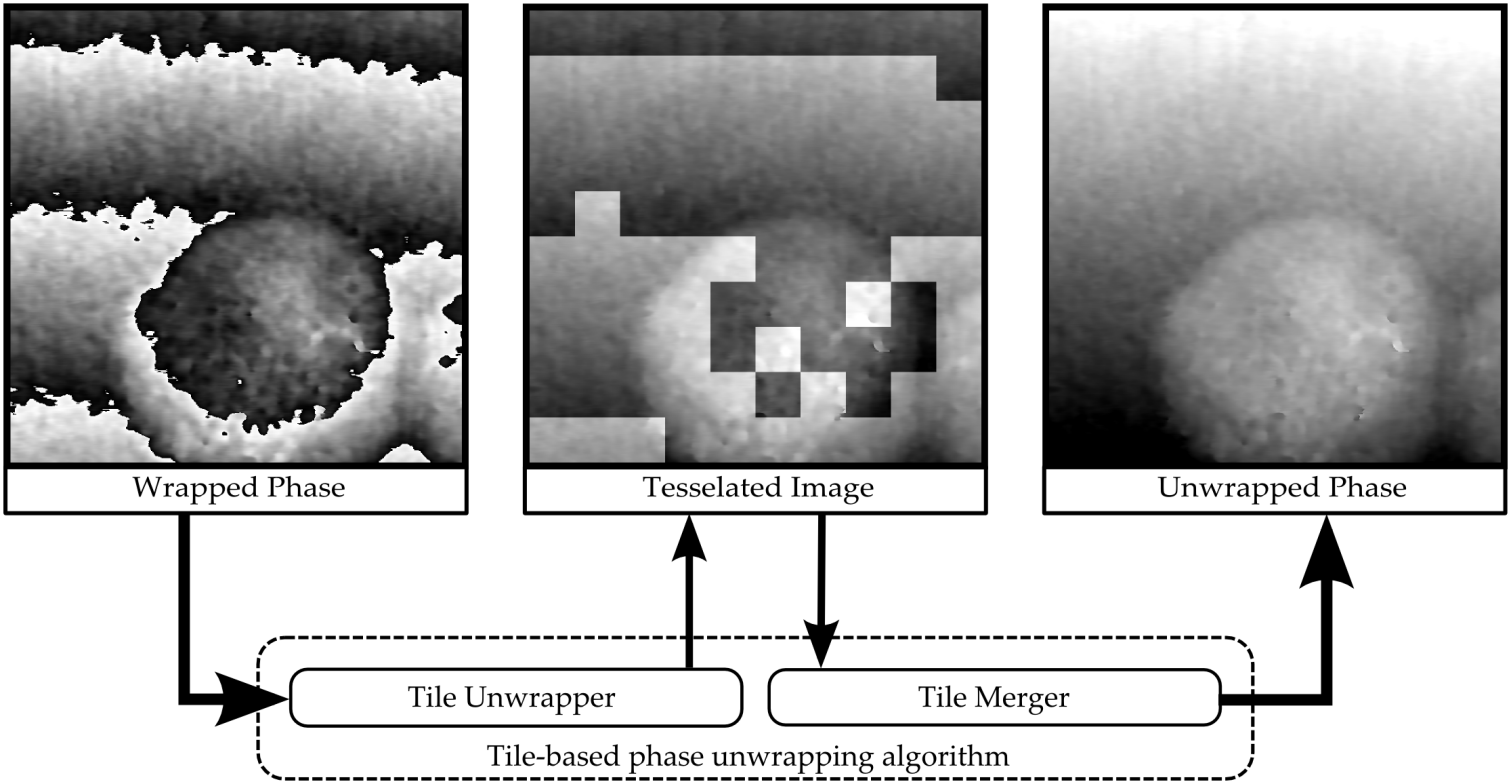
Tile-based phase unwrapping schematic. A wrapped phase image is tessellated into rectangular subregions called tiles. First these tiles are unwrapped individually and second they are merged to a continuous surface.

This process is schematically shown in Fig 1. The total number of tiles for a given tessellation is given as *N*_*τ*_ = *N*_*τW*_ × *N*_*τH*_.

### Model-based phase unwrapping

Model-based phase unwrapping is a type of region based unwrapping, which uses model functions to achieve phase unwrapping on subregions [39–41]. These subregions are merged to a continuous phase map in a second step, which makes it straightforward to adapt it to a tile-based approach. The foundation of model-based phase unwrapping is given by the following fact: Given an arbitrary function *f*(*x*, *y*) it is true that

$$\begin{array}{c} |\phi_{u}\left(x,y\right)-f\left(x,y\right)|<\pi \Rightarrow \phi_{u}\left(x,y\right)=f\left(x,y\right)+W\left(\phi_{w}\left(x,y\right)-f\left(x,y\right)\right). \end{array}$$ (3)

This statement follows from Eq 1, because for any numbers *r* ∈ [−*π*, *π*) and n∈Z it is true that W(r+2πn)=W(r)=r. A way of intuitively understanding Eq 3 is to interpret *f* as a reasonably good guess for the unknown unwrapped phase *ϕ*_*u*_. If such a guess can be obtained from the wrapped phase, then it is possible to calculate the unwrapped phase by adding a correction term $W(\phi_{w}-f)$ to this guess function *f*. In model-based phase unwrapping a model for the guess function is assumed, e.g. polynomial functions [40, 41].

The derivative of the unwrapped phase can easily be obtained from the corresponding wrapped phase map [6]: For a discrete sampling under the Itoh condition it is true that

$$\begin{array}{c} \delta_{x,y}\phi_{u}\left(x,y\right)=W\left(\delta_{x,y}\phi_{w}\left(x,y\right)\right) \end{array}$$ (4)

where *δ*_*x*_ and *δ*_*y*_ are the finite difference operators in *x* and *y* direction respectively. This information can be used to fit a model function to the wrapped phase data [41]. We propose to solve the least squares fitting problem by casting it into a linear system that is solved with singular value decomposition (SVD). This is detailed in the next section.

## Algorithms

### Model-based least squares unwrapping of single tiles (MLSQU)

Unwrapping individual tiles is the first step of the two-step algorithm as described above. Here *ϕ*_*w*_(*x*, *y*) is the discretized wrapped phase distribution given on a tile *τ*_*w*, *h*_ and *ϕ*_*u*_(*x*, *y*) be the corresponding unwrapped phase. The coordinate system (*x*, *y*) belongs to the tile *τ*_*w*, *h*_. Our model-based unwrapping approach is based on Eqs 3 and 4. We make an ansatz for the guess function as a linear combination of *N*_*B*_ base functions *ν*_*l*_

$$\begin{array}{c} f\left(x,y,\vec{c},\rho \right)=\rho +\sum_{l=1}^{N_{B}}c_{l}\cdot \nu_{l}\left(x,y\right) \end{array}$$ (5)

where *ν*_*l*_(*x*, *y*) are analytically given functions, *c*_*l*_ are the expansion coefficients and *ρ* is a constant offset. We assume, without loss of generality, that the constant offset of *f* is solely given by *ρ*. The expansion coefficients are written as a vector $\vec{c}={\left(c_{l}\right)}_{l=1,...,N_{B}}$.

#### Calculating the expansion coefficients

To facilitate the transition to matrix notation we replace the position double index (*x*, *y*) with a single index *k* that enumerates the pixels in a row-major order. Thus we have $k=1,...,N_{px}^{\tau }$, where $N_{px}^{\tau }$ is the number of pixels in a tile. We now write any function *F*(*x*, *y*) as *F*(*k*). A solution for the function expansion coefficients is obtained by least squares fitting the gradient of *f* to the gradient of the unwrapped phase distribution obtained by Eq 4:

$$\begin{array}{c} \chi^{2}\left(\vec{c}\right)=\sum_{k}{∥\vec{\delta }f\left(k,\vec{c}\right)-\vec{\delta }\phi_{u}\left(k\right)∥}_{2}^{2}\to min. \end{array}$$

This minimization is cast into a linear algebra problem by introducing a system matrix $\underline{A}$ and a vector $\vec{g}$ as follows:

$$\begin{array}{ccc} \chi^{2}\left(\vec{c}\right) & = & ∥\underline{A}\cdot \vec{c}-\vec{g}{∥}_{2}^{2}\to min.\\ \underline{A} & = & \left(\begin{array}{c} \underline{A_{x}}\\ \underline{A_{y}} \end{array}\right)\in {ℜ}^{2N_{px}^{\tau }\times N_{B}},\;\text{with}\;{\left(\underline{A_{x,y}}\right)}_{kl}=\delta_{x,y}\nu_{l}\left(k\right)\in {ℜ}^{N_{px}^{\tau }\times N_{B}}\\ \vec{g} & = & \left(\begin{array}{c} {\vec{g}}_{x}\\ {\vec{g}}_{y} \end{array}\right)\in {ℜ}^{2N_{px}^{\tau }},\;\text{with}\;{\left({\vec{g}}_{x,y}\right)}_{k}=W\delta_{x,y}\phi_{w}\left(k\right)\in {ℜ}^{N_{px}^{\tau }} \end{array}$$ (6)

The gradient vector $\vec{g}$ contains the derivatives of the phase distribution within a tile. The system matrix $\underline{A}$ is comprised of the values of the derivatives of the base functions on a tile. Minimization of expression 6 is performed by using the singular value decomposition (SVD) of the system matrix [42]. For a fixed set of base functions, the system matrix only depends on the geometry of a tile. This means that the SVD of the system matrix needs to be calculated only once for any set of tiles with the same width and height. Exploiting this fact is crucial in making this approach computationally feasible. For a tessellation into tiles with identical width and height, unwrapping will be performed with maximum efficiency. Numerical linear algebra was implemented using the Eigen C++ library [43].

#### Calculating the constant offset

Considering Eq 5 suggests determining *ρ* by subtracting the value of the linear combination of base functions with known expansion coefficients from the value of the tile at an arbitrary point (*x*_0_, *y*_0_). This approach is feasible in low noise settings and for applications where additional computational overhead is not desired. For maximum robustness against noise, we decided to incorporate Strand’s tile unwrapping algorithm into our procedure [29]. Accordingly, we define a penalty function Π(*τ*) for a tile *τ*:

$$\begin{array}{c} \Pi \left(\tau \right)=\langle ∥\delta_{x}^{1}\phi_{w}{\left(x,y\right)∥}_{2}\rangle +\langle ∥\delta_{y}^{1}\phi_{w}\left(x,y\right){∥}_{2}\rangle \end{array}$$ (7)

where $\delta_{x,y}^{1}$ are the first order forward difference operators and 〈.〉 signifies the average over all positions in the tile *τ*. Note that the application of the finite difference operator to the wrapped phase is not followed by a wrap operation, so that phase jumps will contribute with 2*π* to the penalty function. Thus this function is a measure for the number of phase jumps within a tile. First we calculate

$$\begin{array}{c} \phi_{rem}\left(x,y\right):=W\left(\phi_{w}\left(x,y\right)-f\left(x,y,\vec{c},\rho =0\right)\right). \end{array}$$

This is the remainder of the phase after the removal of the surface given by the model function *f* with *ρ* = 0. Next the constant *ρ* is calculated as

$$\begin{array}{c} \rho =\underset{\rho^{\prime }\in \left[0,2\pi \right)}{\text{arg min}}\Pi \left(W\left(\phi_{d}\left(x,y\right)-\rho^{\prime }\right)+\rho^{\prime }\right). \end{array}$$ (8)

This is the application of Strand’s unwrapping approach to the remainder of the phase. A detailed description and an intuitive interpretation of Eqs 7 and 8 is found in [29]. Minimization of Eq 8 is performed by evaluating the penalty function at *N*_*ρ*_ equidistantly spaced values for *ρ* ∈ [0, 2*π*). Finally, using Eq 3, the model-based tile unwrap is performed using the constant *ρ* as calculated above. Our tile unwrapping approach thus contains Strand’s tile unwrapping method as a special case for a model function *f*(*x*, *y*) = *const*. = *ρ*.

### Merging Algorithms

After the unwrap of all individual tiles is completed, neighboring tiles can show phase jumps of integer multiples of ±2*π* or 0 with respect to each other. A merging algorithm, or *merger*, joins the tiles of a tessellated image to a continuous phase surface by adding integer multiples of ±2*π* to entire tiles. Adding a value to a tile means adding this value to every pixel within the tile.

We term the set of neighboring pixels for two tiles *τ*_*A*_, *τ*_*B*_ the *junction* $J_{AB}$. The set of differences between neighboring pixels for a junction is introduced as

$$\begin{array}{c} D\left(J_{AB}\right):=\left\{\left(P_{B}-P_{A}\right)|P_{A}\in \tau_{A},P_{B}\in \tau_{B}\;\text{are}\;\text{neighbor}\;\text{pixels}\right\}. \end{array}$$

Since the phase distribution is tessellated into a rectangular grid, any tile at coordinates *A* = (*w*_*A*_, *h*_*A*_) can have up to four neighbors, corresponding to the directions up (*w*_*A*_, *h*_*A*_ − 1), down (*w*_*A*_, *h*_*A*_ + 1), left (*w*_*A*_ − 1, *h*_*A*_) and right (*w*_*A*_ + 1, *h*_*A*_). To quantify the phase jumps between neighboring tiles, we introduce the mean difference $\Delta J_{AB}$ and the variance $VarJ_{AB}$ of a junction as

$$\begin{array}{ccc} \Delta J_{AB} & := & Mean(D\left(J_{AB}\right))\\ VarJ_{AB} & := & Variance(D\left(J_{AB}\right)) \end{array}$$

Algorithm 1 unwraps two neighboring tiles with respect to each other by using the mean difference of the corresponding junction. Algorithms in this paper are given in pseudocode notation.

**Algorithm 1** Merge tile *τ*_*A*_ to tile *τ*_*B*_

1: **Procedure** merge tiles (*τ*_*A*_, *τ*_*B*_)

2:  ▹ Find the mean difference of neighboring pixels:

3:  delta $\leftarrow \Delta J_{AB}$

4:  ▹ Round difference to nearest integer multiple of ±2*π*:

5:  jumpval ←2*π*⋅ round (delta/2*π*)

6:  **Add** jumpval to all pixels of *τ*_*A*_

#### Unidirectional tile merger

In [29], Strand *et al.* suggest a simple and fast merging algorithm that requires only one pass over each tile, see Algorithm 2. It is a modification of Itoh’s classic one dimensional unwrapping approach [6].

**Algorithm 2** Unidirectional tile merger (column-wise)

1: ▹ Go through tiles column-wise left to right:

2: **for** *iw* ← 1, …, *N*_*τW*_ **do**

3:  ▹ Go through each column of tiles from top to bottom:

4:  **for** *j* ← 1, …, *N*_*τH*_ **do**

5:   ▹ Find top and left neighbors of current tile *τ*_*iw*, *ih*_, if they exist:

6:   neighbors ← {*τ*_*iw* − 1, *ih*_, *τ*_*iw*, *ih* − 1_} ∩ {*τ*_*w*, *h*_|*w* = 1, …, *N*_*τW*_, *h* = 1, …, *N*_*τH*_}

7:   ▹ Find mean value of junction differences to neighbours:

8:   delta ← mean value of junction differences of tile *τ*_*iw*, *ih*_ to neighbors

9:   ▹Round difference to nearest integer multiple of ±2*π*:

10:   jumpval ←2*π* ⋅ round(delta/2*π*)

11:   **Add** delta to all pixels of *τ*_*iw*, *ih*_

#### Reliability guided SRNCP merger (*τ*SRNCP)

We developed a reliability guided tile merger based on the SRNCP (*“sorting by reliability following a non-continuous path”*) algorithm by Herráez *et al.* [10]. The fundamental principle of the pixel-based SRNCP algorithm is to analyze the junctions of neighboring pixels and unwrap them sequentially according to a reliability value. Junctions with the highest reliability are processed first and unwrapped pixels are grouped to identify sets of pixels with no remaining phase jumps between each other.

The reliability of the pixel-based SRNCP algorithm makes use of the second derivative of the phase map. This quantity cannot simply be generalized to tiles. To adapt this algorithm to a tile-based approach, we introduce a new reliability measure for junctions and tiles, respectively. The reliability of a tile is calculated as follows: First, calculate the variance $VarJ_{AB}$ of all junctions of this tile and assign its average value to *V*. The reliability of the tile is defined as *R* = *V* ^−1^. This means that a tile has high reliability, when it segues into all of its neighbors continuously. This is also true if the tiles differ by multiples of 2*π* along the junction, since the variance is invariant under a constant offset. Noise and phase residues, however, will decrease the reliability. The reliability of a junction is defined as the product of the reliabilities of the two tiles belonging to it. The process of grouping and unwrapping tiles with respect to each other is analogous to the pixel-based SRNCP and detailed in Algorithm 3.

It is by the definition of the reliability that our tile-based SRNCP merger substantially differs from the pixel-based SRNCP algorithm. Our definition of reliability is meaningful only for a tile-based approach and does not converge to the pixel-based method in the limit of single pixel tiles. Whenever we speak of the pixel-based SRNCP algorithm in this paper we refer to the original implementation of Herráez *et al.*, while the tile-based *τ*SRNCP merger refers to our adaption of a merging algorithm.

**Algorithm 3** Tile-based *τ*SRNCP merger

Calculation of junction reliabilities

1: L← List of all junctions in the tessellated image.

2: **calculate** reliability for each junction in L.

Grouping and merging tiles

3: **set** each tile as belonging to no group.

4: **sort** L in descending order of junction reliability.

5: **for all** junctions $J_{AB}\in L$ **do**

6:  **if** neither *τ*_*A*_ nor *τ*_*B*_ belong to a group **then**

7:   mergetiles (*τ*_*A*_, *τ*_*B*_) and generate a new group containing both tiles.

8:  **else if** only *τ*_*B*_ has a group **then**

9:   mergetiles (*τ*_*A*_, *τ*_*B*_) and add *τ*_*A*_ to group of *τ*_*B*_.

10:  **else if** only *τ*_*A*_ has a group **then**

11:   mergetiles (*τ*_*B*_, *τ*_*A*_) and add *τ*_*B*_ to group *G*_*A*_.

12:  **else**      ▹ Both tiles belong to group

13:   Merge all tiles of smaller group to the tile *τ*_*A*, *B*_ with the larger group.

14:   Assimilate the smaller group in the larger group.

## Methods

To assess the quality of the proposed method for synthetic and measured datasets, we have evaluated different combinations of tile unwrappers and mergers. We have further analyzed the performance of the pixel-based SRNCP algorithm. For model-based tile unwrapping we chose monomial base functions, so the resulting model function *f* on every tile can be written as

$$\begin{array}{c} f\left(x,y\right)=\rho +\sum_{i=0,...,P_{x}}\;\sum_{j=0,...,P_{y}}c_{ij}\cdot x^{i}y^{j} \end{array}$$

where *P*_*x*_ and *P*_*y*_ are the maximum degrees of powers in *x* and *y* respectively. We set *P*_*x*_ = *P*_*y*_ = *P*, resulting in a polynomial of degree 2*P* and in a number of (*P* + 1)^2^ − 1 coefficients. The term *x* ^0^ *y* ^0^ is absorbed into the constant offset *ρ*.

### Application to synthetic phase maps with noise

A ground-truth synthetic phase map *ϕ*_*u*_ was generated on a quadratic raster with *N*_*X*_ = *N*_*Y*_ = 600 pixels. The top left pixel of this raster is designated as the origin (0, 0). The synthetic phase consists of two overlapping Gaussian profiles and is given analytically by

$$\begin{array}{c} \phi_{u}\left(x,y\right)=4\pi \cdot \exp\left(-\frac{{\left(x-\frac{N}{3}\right)}^{2}+{\left(y-\frac{N}{3}\right)}^{2}}{2\cdot {\left(6N\right)}^{2}}\right)+8\pi \cdot \exp\left(-\frac{{\left(x-\frac{2N}{3}\right)}^{2}+{\left(y-\frac{2N}{3}\right)}^{2}}{2\cdot {\left(8N\right)}^{2}}\right). \end{array}$$

While the particular shape of the profile was chosen arbitrarily, Gaussian phase profiles are often used as synthetic phase distributions [16, 44, 45]. Furthermore the Gaussian phase profile cannot simply be fitted by a set of monomial base functions. We obtained a noisy phase distribution $\phi_{u}^{\sigma }\left(x,y\right)=\phi_{u}\left(x,y\right)+N_{\sigma }\left(x,y\right)$ by adding a zero mean white Gaussian noise distribution $N_{\sigma }\left(x,y\right)$ with standard deviation *σ*. Noisy wrapped phase distributions were generated using $\phi_{w}^{\sigma }=W\phi_{u}^{\sigma }$ for *σ* ∈ [0, 1] in steps of 0.1. Wrapped phase maps were processed with different combinations of tile-based unwrapping and merging algorithms as well as the pixel-based SRNCP algorithm.

To quantify the fidelity of the result obtained by an algorithm $\phi_{u}^{alg}$ to the ground truth phase $\phi_{u}^{\sigma }$, we calculated the *deviation* *D* of the ground truth and unwrapped phase maps

$$\begin{array}{c} D=\frac{1}{N_{X}N_{Y}}\sqrt{\sum_{x,y}{\left|\phi_{u}^{alg}\left(x,y\right)-\phi_{u}^{\sigma }\left(x,y\right)-\mu \right|}^{2}} \end{array}$$

with $\mu =\langle \phi_{u}^{alg}-\phi_{u}^{\sigma }\rangle$ to compensate for a possible constant offset between these phase maps. The mean deviation of 25 distributions for each Gaussian noise level *σ* was calculated and plotted as a function of the noise level *σ*.

Adding zero mean white Gaussian noise to phase images is a common practice to quantify the performance of phase unwrapping algorithms [7, 16, 29, 46]. It must be noted, however, that zero mean white Gaussian noise is not a valid model for many noise sources in real world applications [29, 47, 48]. Yet, the results give an accurate indication of the robustness of the algorithm against violations of the Itoh sampling condition due e.g. to low signal to noise ratio of measured datasets [49].

### Application to a synthetic phase map with noise and residues

The performance of an unwrapping algorithm in the presence of residues is an important issue in many applications [21, 22, 50]. To qualitatively gauge the performance of the tile-based approach presented here in the presence of phase residues, we generated a synthetic phase map as follows: First we created a noiseless ground truth distribution as detailed in the previous section. Next, we placed a rectangle over a phase wrap line and applied a Gaussian blur filter with a width of 5 pixels. This creates a smooth border and removes the phase jump within this area. Finally, we added a zero mean Gaussian noise distribution with *σ* = 0.3 to the entire phase map and wrapped the resulting image to obtain a noisy wrapped phase with residues. Since there is no unique solution to unwrapping any wrapped phase distribution with residues, we compared the performance of several tile-based algorithms and the pixel-based SRNCP unwrapper by qualitatively assessing the influence of the phase residue on the result.

#### Application to measured digital holographic datasets

The proposed algorithm was also applied to real world wrapped phase distributions that were obtained with a digital holography setup as reported by Kalies and Antonopoulos *et al.* [51]. Trypsinized ZMTH3 canine adenoma cells, kindly provided by Murua Escobar *et al.*, served as a sample [52]. The wrapped phase map has a dimension of 1400 × 1400 pixels.

## Results and Discussion

### Synthetic phase with noise

**Fig 2:**
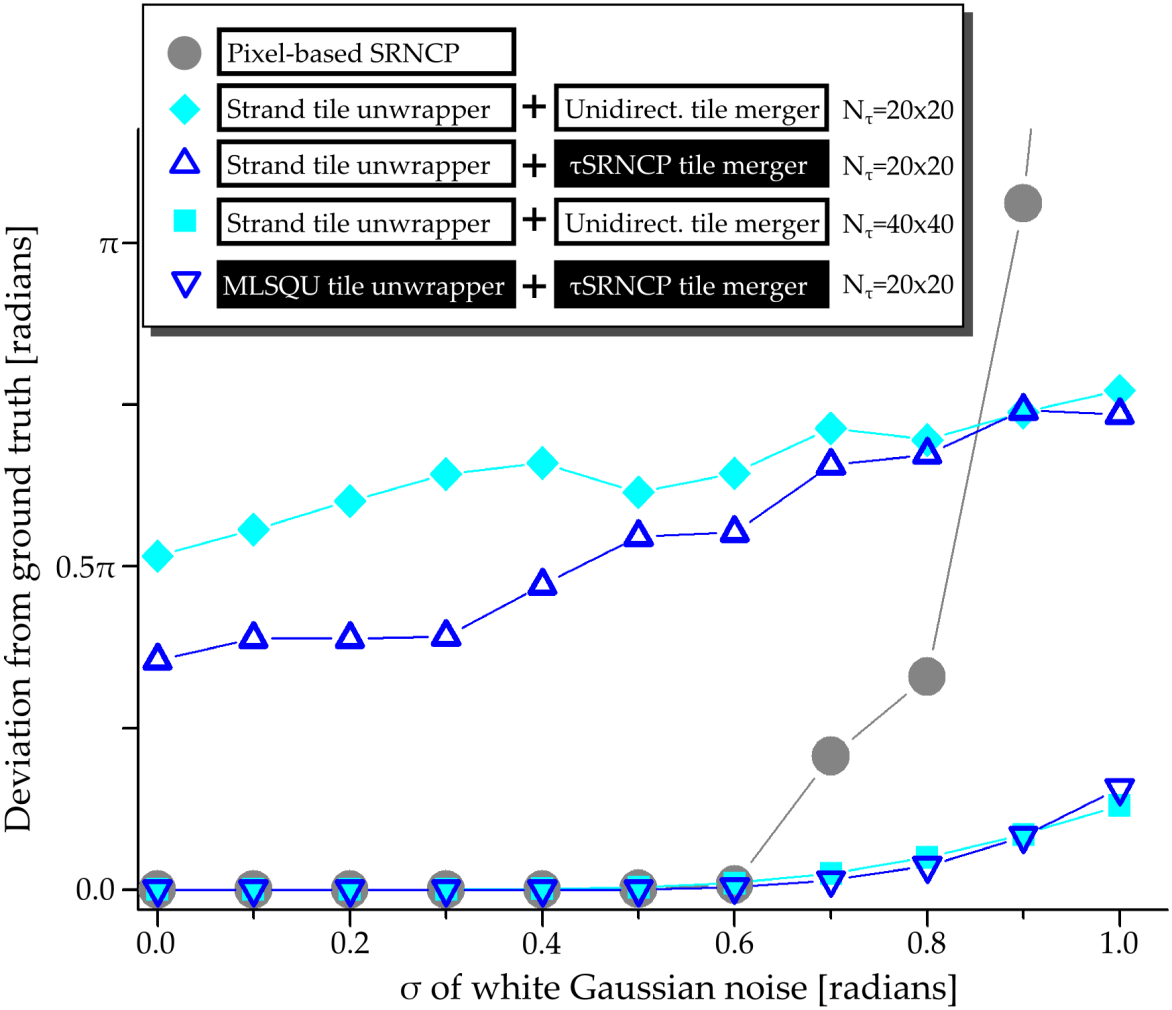
Deviation of the unwrapped phase from a noisy ground truth for different algorithms. The mean deviation of 25 phase images to the ground truth with a white Gaussian noise of specified *σ* is shown. (▿) The proposed algorithm using a model-based tile unwrapper with polynomial base *P* = 2 and *N*_*τ*_ = 20 × 20 tiles and *N*_*ρ*_ = 40 steps for minimization. Merging was performed using our *τ*SRNCP merger. (•) The pixel-based SRNCP algorithm. At *σ* = 1.0 the deviation is outside the range of the plot with a value of 7.7 radians. (■) Strand’s original algorithm using a tessellation into *N*_*τ*_ = 40 × 40 tiles and *N*_*ρ*_ = 20. The plots for (✦) and (△) show the results of using Strand’s unwrapper with a tile size containing more than one phase wrap using *N*_*ρ*_ = 40. In this case, the unwrapping of those tile fails and the deviation is analyzed for two different merging algorithms: (✦) the unidirectional merger and (△) the *τ*SRNCP merger. Algorithms proposed in this paper are in black boxes.

**Fig 3:**
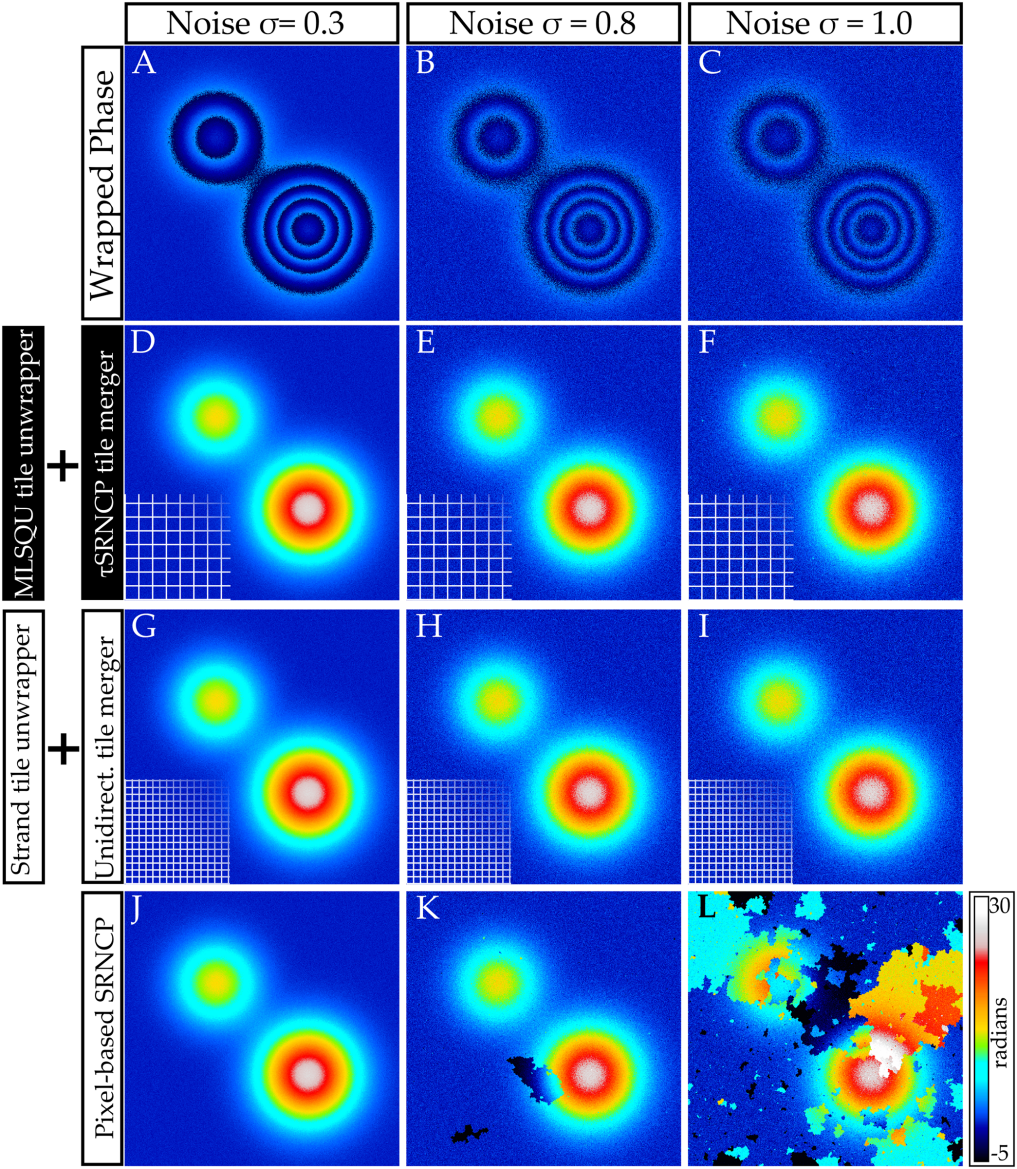
Comparison of phase unwrap with different algorithms for various noise levels. **A,B,C:** Wrapped phase distributions for different noise levels. **D,E,F:** Tile-based unwrap with a tessellation into 20 × 20 tiles. Tile unwrapping was performed with the model-based least squares unwrapper (MLSQU) using *P* = 2, *N*_*ρ*_ = 40. Merging was performed with the tile-based *τ*SRNCP merger. **G,H,I:** Tile-based unwrap with a tessellation into 40 × 40 tiles. Tile unwrapping was done with Strand’s unwrapper (*N*_*r*_ *ho* = 20) and a unidirectional merger. This corresponds to Strand’s original algorithm. **J,K,L:** Unwrapped phase map using the pixel-based SRNCP algorithm. For a full resolution graphic see S1 Fig. Algorithms proposed in this paper are in black boxes.

We analyzed the deviation of the unwrapped phase from the noisy ground truth as a function of different noise levels *σ* for various algorithms (see Fig 2 for the data and Fig 3 for exemplary images). Deviations were calculated by averaging over 25 phase maps for each noise level. We compared different combinations of tile-based unwrapping and merging algorithms with the pixel-based SRNCP unwrapper. We also reproduced Strand’s original unwrapping algorithm by combining Strand’s tile unwrapper and the unidirectional merger. For Strand’s algorithm we used a tessellation into *N*_*τ*_ = 40 × 40 tiles. Our proposed algorithm was tested by tessellating the phase into *N*_*τ*_ = 20 × 20 tiles and applying our model-based tile unwrapper with polynomial order *P* = 2. For merging we used our *τ*SRNCP merger. Up to a noise level of *σ* = 0.3, these three phase unwrapping algorithms yield very good results with average deviations of the order of 10^−5^. The mean deviation rises to the order of 10^−2^ up to a noise level of *σ* = 0.6. For higher noise levels the deviations rise to 0.4 and 0.49 at *σ* = 1.0 for Strand’s algorithm and the proposed algorithm respectively. In contrast, a major rise of the deviation (7.5 at *σ* = 1.0) is observable in the case of the pixel-based SRNCP merger. This is caused by propagation of unwrapping failures of single pixels to large areas. For the tile-based approaches, erroneous unwraps remain within tiles and are much less likely to be propagated to further tiles in the merging step. This is the reason for the robustness of the tile-based approach to high frequency noise. Note that a tile-based *τ*SRNCP merger also inherits this robustness while still operating on the reliability guided merging principle of the original algorithm.

We explored the influence of different merging algorithms on the result by testing it in a case where the unwrap of several tiles fails. For this, tiles were unwrapped with Strand’s algorithm using a tessellation into *N*_*τ*_ = 20 × 20 tiles. Some tiles contained more than one phase wrap, so that they could not be correctly unwrapped even in the noise-free case. For noise levels below *σ* = 0.8, the *τ*SRNCP produced lower deviation from the ground truth than the unidirectional merger. This is due to a tendency of the unidirectional merger to propagate unwrap failures to subsequent tiles, see S1 Fig. The *τ*SRNCP merger is much less likely to propagate errors to large areas since it processes reliable junctions first. This demonstrates, that employing a quality guided merger can reduce the influence of failed tile unwrapping on the result compared to a linear approach. For reference, runtimes of the algorithms used in this section are given in Table 1. Due to the computational overhead introduced by the framework and the complexity of linear algebra operations, algorithms involving the MLSQU tile unwrapper have longer execution times compared to simpler algorithms. It has to be noted, however, all unwrapping processes were executed sequentially on a single core, since no parallelization has been implemented, yet.

**Table 1:** Runtimes of phase unwrapping algorithms. For tile-based algorithms the total runtime is the sum of the runtimes of tile unwrapper and tile merger. For the model-based tile unwrapper (MSLQU) the precalculation of the system matrix has to be performed once and is given in braces. A synthetic noisy distribution with *σ* = 0.6 and dimensions of 600×600 pixels was unwrapped in all cases. Runtimes were measured on a high-end desktop PC (Intel^®^ i5-4570 3.2GHz CPU, 32 GB RAM) with sequential execution on a single core. Runtimes are given as the mean of five independent measurements.

Tesselation *N*_*τ*_	Algorithm	Runtime
40 × 40	Strand tile unwrapper (*N*_*ρ*_ = 40)	453.0 ms
	Unidirectional tile Merger	3.0 ms
20 × 20	Strand tile unwrapper (*N*_*ρ*_ = 20)	236.4 ms
	Unidirectional tile merger	1.0 ms
20 × 20	Strand tile unwrapper (*N*_*ρ*_ = 20)	235.0 ms
	*τ*SRNCP tile merger	3.4 ms
20 × 20	MLSQU tile unwrapper (*N*_*ρ*_ = 20, *P* = 2)	578.8 ms (+1.6 ms)
	*τ*SRNCP tile merger	4.0 ms
2 × 2	MLSQU tile unwrapper (*N*_*ρ*_ = 60, *P* = 2)	1342 ms (+1533 ms)
	*τ*SRNCP tile merger	< 1 ms
none	Pixel-based SRNCP unwrapper	155.4 ms

### Synthetic phase with noise and residues

**Fig 4:**
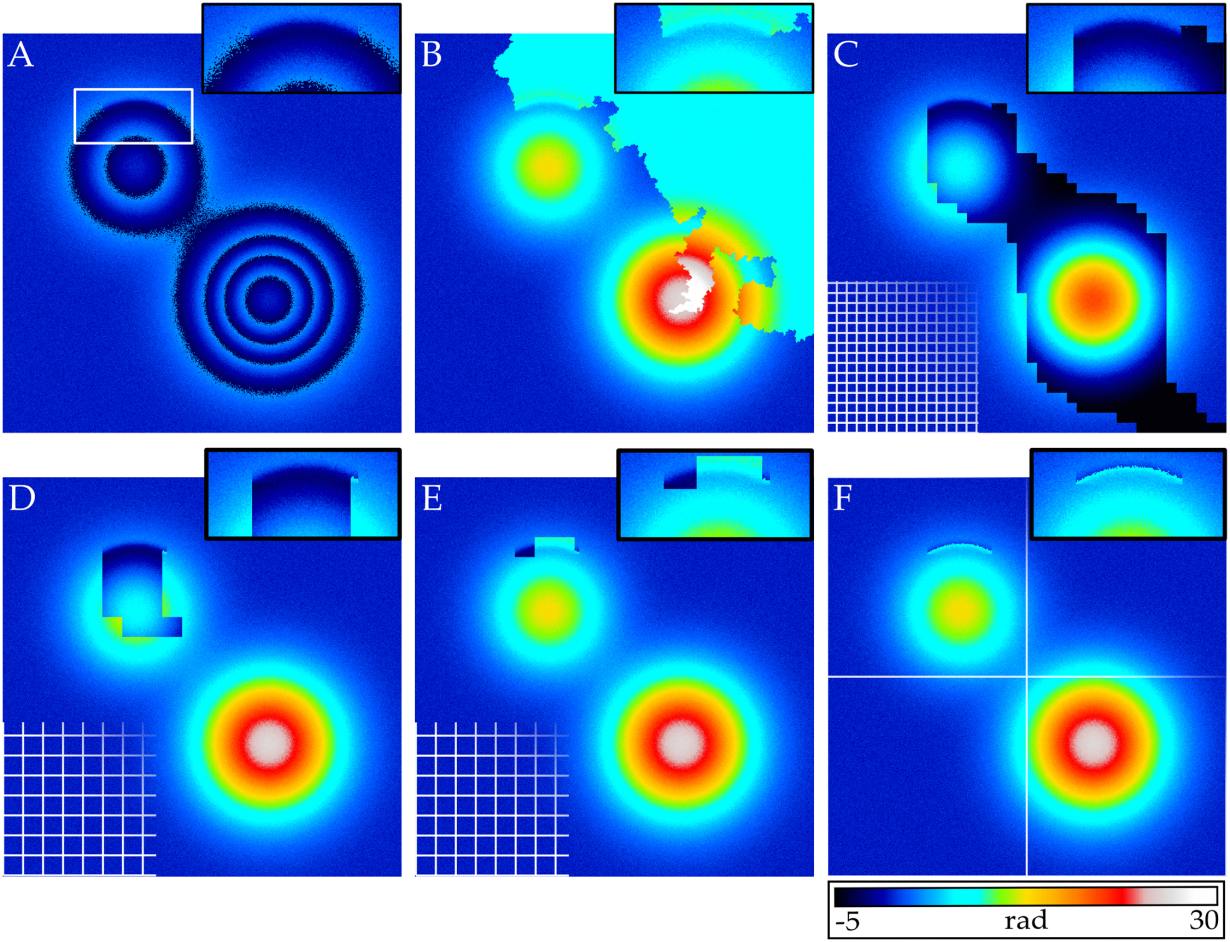
Unwrap of a synthetic phase map with noise and residues. **A:** Wrapped phase distribution with a noise level of *σ* = 0.3. The residue is indicated by an arrow and the a close-up of the surrounding area is shown in an inset. **B:** Pixel-based SRNCP unwrap. **C:** Tilebased unwrap with *N*_*τ*_ = 40 × 40 tiles using Strand’s tile unwrapper (*N*_*ρ*_ = 20) and Strand’s unidirectional merger. **D:** Unwrapped phase using the proposed MLSQU tile unwrapper with *P* = 2, *N*_*ρ*_ = 40 and the unidirectional tile merger. Tile count is *N*_*τ*_ = 20 × 20, resulting in double the tile size compared to C. **E:** Same as D, but using the proposed tile-based *τ*SRNCP algorithm for merging. **F:** Unwrapped phase using MLSQU tile unwrapper with *P* = 6, *N*_*ρ*_ = 60 and the tile-based *τ*SRNCP merger. The image was tessellated into a 2 × 2 grid. All images have dimensions of 600 × 600 pixels and the same color scale as Fig 2.

Results of unwrapping a synthetic phase map with noise and residues are shown in Fig 4. We unwrapped the phase map with the pixel-based SRNCP algorithm (Fig 4B) and Strand’s original unwrapping and merging algorithm (Fig 4C). For Strand’s algorithm we used a tile count of *N*_*τ*_ = 40 × 40 tiles in x- and y-directions. Both algorithms propagate unwrapping errors over approximately a third and a quarter of the image, respectively. In the synthetic noisy case without residues, both algorithms were shown to yield very good results for up to double the noise level. Next, we compared the results of combining the proposed model-based tile unwrapper with Strand’s unidirectional merger as well as our proposed *τ*SRNCP merger. A polynomial model with *P* = 2 was used and the tile count set to *N*_*τ*_ = 20 × 20, thereby doubling the area of each tile compared to Fig 4C. Merging the tiles with the unidirectional merger propagates unwrapping errors due to the phase residue to a number of neighboring tiles, see Fig 4D. When the tiles are merged with the *τ*SRNCP merger, the unwrapped phase is affected only on the four tiles containing the residue. Since all four tiles are affected by the phase residue at one or more borders, their reliability is decreased compared to tiles with no residues. This ensures that they will be processed last in the merging process. Thus, unwrapping errors are not propagated to more tiles. The affected area corresponds to 0.25% of the total area of the phase map. This shows, that combining model-based tile unwrapping with a reliability guided merging process can prevent the propagation of unwrapping errors in an unwrapped phase map in the case where residues are present.

Finally, we tessellated the image into merely 2 × 2 tiles and unwrapped the tiles with the MLSQU tile unwrapper using a polynomial with *P* = 6, see Fig 4F. The residue is located within the top left tile and after unwrapping the residue has minimal impact on the tile. Merging is performed using the *τ*SRNCP merger, but using the unidirectional merger will produce the same result since the residue does not affect the border of the tile. Thus, by choosing the size of the tiles large enough that residues are located within the tiles, the final unwrapped phase will only be affected on pixels close to the residue. This demonstrates the value of our model-based tile unwrapper compared to Strand’s method: the proposed model-based tile unwrapping algorithm is capable of processing tiles with more than just one phase wrap inside.

In summary, we have shown three key points on synthetic datasets: First, a tile-based approach increases the robustness of the reliability guided SRNCP unwrapping algorithm against high frequency noise. Second, the *τ*SRNCP merging algorithm can significantly increase the quality of an unwrapped phase map when tile unwrapping fails due to noise or residues. Finally, the model-based tile unwrapping algorithm proposed in here allows for increased tile size compared to Strand’s approach. The unwrapper performs well in the presence of noise and can be beneficial in the presence of phase residues. On a more general level, we believe that this validates our approach of creating a modular tile-based phase unwrapping concept that works with different combinations of unwrapping and merging algorithms.

### Digital holographic phase measurements

**Fig 5:**
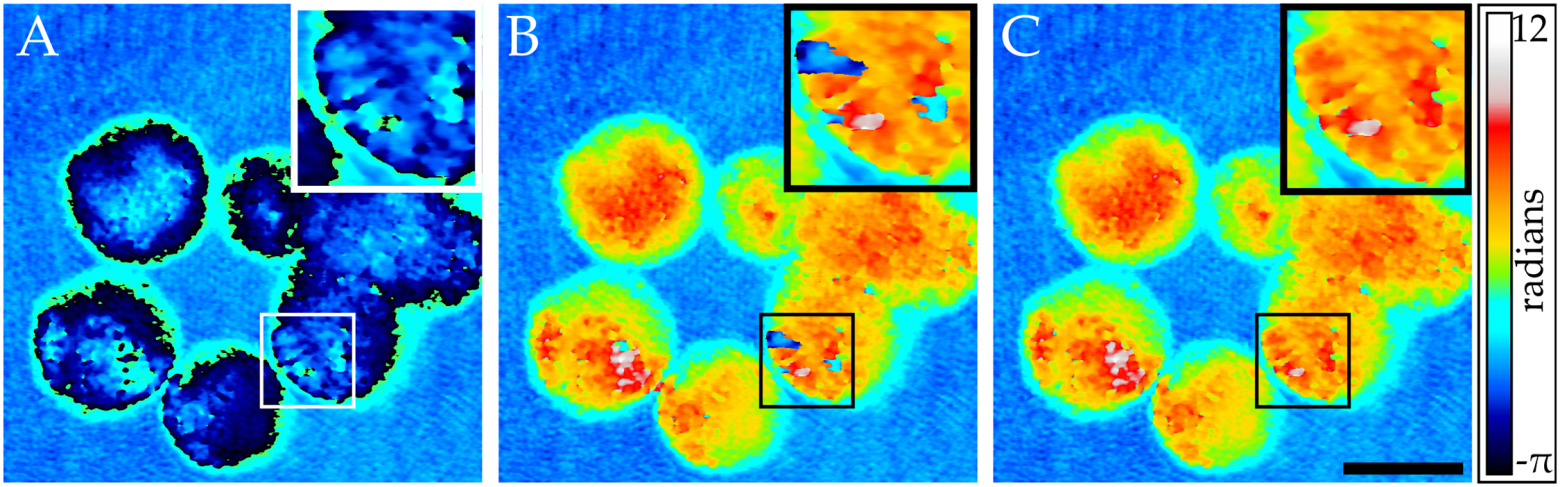
Phase unwrap of ZMTH3 canine adenoma cells captured with digital holography. **A:** Wrapped phase map. **B:** Pixel-based unwrap with the SRNCP algorithm. **C:** Unwrap using the proposed approach by tessellating the image into 8 × 8 tiles and unwrapping individual tiles with the model-based approach using polynomial order *P* = 4. Tiles were merged with the unidirectional merger. A magnification of a region with phase residues is shown. The phase maps consist of 1400 × 1400 pixels. Scale bar is 15 *μ* *m*.

We applied our phase unwrapping algorithm to a wrapped phase image of ZMTH3 canine adenoma cells obtained with digital holography, see Fig 5. The wrapped phase map contains multiple residues but it is free of high frequency noise. We compared the phase unwrap of the pixel-based SRNCP algorithm with our tile-based approach using a model-based unwrapper (*P* = 2, *N*_*ρ*_ = 40) and a unidirectional merger. Both algorithms appear to be successful in unwrapping the given phase distribution and do not produce obvious inaccuracies. Upon closer inspection, however, the pixel-based unwrap shows several areas in which unwrapping errors due to phase residues were propagated to larger regions. Our tile-based approach performs more robustly in the presence of residues in accordance with the results from the previous section.

## Conclusion

We have presented a modular tile-based phase unwrapping approach that was cast into a C++11 program with a modular framework. In a two step process, individual tiles are unwrapped first and then merged to an unwrapped phase map using a merging algorithm. A set of tile unwrapping and merging algorithms was implemented as suggested by Strand *et al*. We developed a model-based unwrapper that contains Strand’s unwrapper as a special case. Both algorithms performed well in synthetic high noise scenarios. We could further show that our model-based approach is superior to Strand’s original unwrapping algorithm in scenarios with phase residues due to its ability to process larger tiles. This was demonstrated for a synthetic wrapped phase map as well as a real world phase map obtained from digital holography of canine adenoma cells. Model-based tile unwrapping was formulated as a least squares minimization using linear algebra. As a next step this could allow use of parallelization to speed up the unwrapping process significantly, since linear algebra lends itself nicely to parallel computing. In this study we did not implement parallelization and can therefore not take full advantage of this approach, yet.

We could further create a reliability guided tile merger from the pixel-based SRNCP algorithm. This so called *τ*SRNCP merger performed superior compared to a linear merger in scenarios with erroneous unwraps as well as phase residues. It also performed superior to the pixel-based SRNCP algorithm in the presence of noise and residues. We believe that this validates the idea of the modular tile-based phase unwrapping concept behind our software framework. It also indicates that adapting well-established pixel-based algorithms to a tile-based approach is possible and worth-while. The computational load on these merging algorithms is significantly decreased to their pixel-based counterparts, because they operate on the tiles of the tessellated image instead of single pixels. This fact could make the implementation of more complex algorithms, such as optimization based on simulated annealing, computationally feasible for large phase maps.

Typically, the choice of phase unwrapping algorithm is—to an extent—dependent on the properties of wrapped datasets themselves, e.g. the level of noise. Furthermore there might be user requirements that favor a fast algorithm over one with high robustness against noise. It is obviously false, that a slower, more complex algorithm will generally result in better unwrapped phase maps. However, there is a trade-off between speed and robustness. We feel that the proposed framework offers an advantage that might make it interesting for some applications: The modular framework makes it possible to tailor unwrapping algorithms to specific needs. It serves as an easily extensible toolbox that can be used to test and create new tile-based phase unwrapping algorithms before committing to an individual algorithm. It might further be possible to perform the tailoring process automatically. For that, a sufficiently large set of unwrapping and merging algorithms is needed. Then it is conceivable that the particular combination of unwrapper and merger can be constructed automatically using machine learning with appropriate training sets. One example of a specially tailored algorithm is a combination of the Strand tile unwrapper and the unidirectional merger using a parallel implementation. This could be fast enough for live imaging, if a reduced image quality is acceptable. Another example stems from the properties of the MLSQU tile unwrapping algorithm: Here the precalculation of the system matrix makes it possible to apply high order polynomial fits to large tiles. As a consequence, high numbers of images can be processed efficiently. While the computational load is likely still rather high compared to other algorithms, a possible gain in image quality could make up for this. By making our software publicly available we hope that other researchers may find it useful to use and improve upon it.

## Supporting Information

S1 Fig Full resolution version of Fig 3. Additional rows show results for tessellation of the original image into 20 × 20 and using Strand’s unwrapper (*N*_*ρ*_ = 40) for individual tiles. In this case, the unwrap of all tiles that contain more than one phase wraps fails. Merging is performed using two different algorithms: **M,N,O:** Unidirectional Merger and **P,Q,R:** tile-based *τ*SRNCP merger. Algorithms proposed in this paper are in black boxes.
(TIF)

S2 Fig Schematic illustration of phase residues. **A** Wrapped phase image of a silica microsphere in water. At the location of the arrows a so-called fringe washout is visible. This smoothes the phase, such that in a wrapped phase no phase jumps will be detectable. **B** Schematic of the isolines of 2*π* phase jumps between adjacent pixels. At the position of the fringe washout the isolines are discontinued. These open isolines are errors of the measurement and do not represent physical reality. For an error-free wrapped phase map of a physical phase, all isolines inside the area of measurement must be closed. The tips of open isolines are so called residues. A closed loop (*C*_1_) around such a residue will count a different number of positive and negative phase jumps. In contrast, any closed loop (*C*_2_) around a closed isoline will count the same number of positive and negative phase jumps. This illustration given here is rather intuitive and it suffices for the purposes of this study. For a more technical definition refer to [19].
(TIF)
