# Supplementary figures and images for: Tile-Based Two-Dimensional Phase Unwrapping for Digital Holography Using a Modular Framework
Source: PLoS One. 2015 Nov 24;10(11):e0143186. doi: 10.1371/journal.pone.0143186 (PMC4657957; doi:10.1371/journal.pone.0143186)

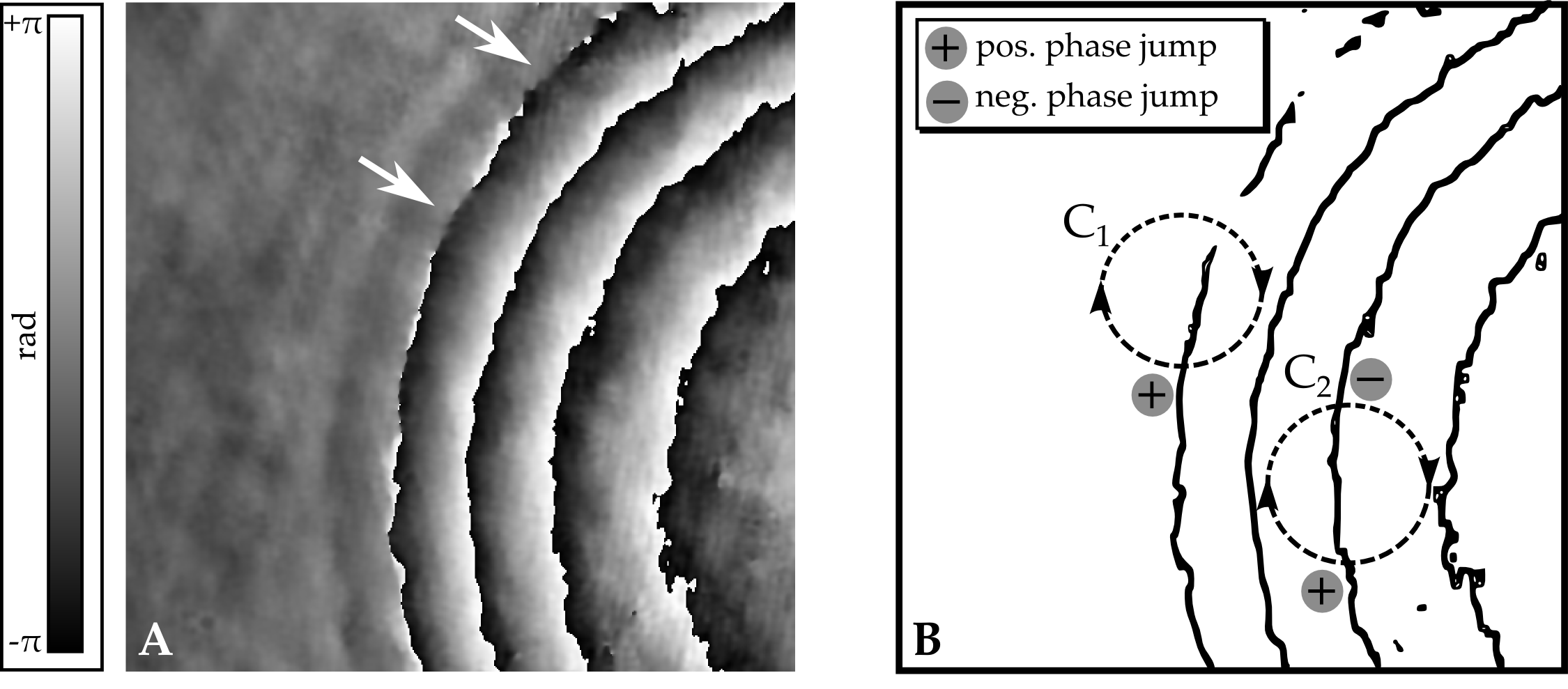

Supplement: S2 Fig — A Wrapped phase image of a silica microsphere in water. At the location of the arrows a so-called fringe washout is visible. This smoothes the phase, such that in a wrapped phase no phase jumps will be detectable. B Schematic of the isolines of 2π phase jumps between adjacent pixels. At the position of the fringe washout the isolines are discontinued. These open isolines are errors of the measurement and do not represent physical reality. For an error-free wrapped phase map of a physical phase, all isolines inside the area of measurement must be closed. The tips of open isolines are so called residues. A closed loop (C 1) around such a residue will count a different number of positive and negative phase jumps. In contrast, any closed loop (C 2) around a closed isoline will count the same number of positive and negative phase jumps. This illustration given here is rather intuitive and it suffices for the purposes of this study. For a more technical definition refer to [19]. (TIF) [file pone.0143186.s002.tif]
